# Of Rings and Rods: Regulating Cohesin Entrapment of DNA to Generate Intra- and Intermolecular Tethers

**DOI:** 10.1371/journal.pgen.1006337

**Published:** 2016-10-27

**Authors:** Robert V. Skibbens

**Affiliations:** Department of Biological Sciences, Lehigh University, Bethlehem, Pennsylvania, United States of America; Harvard Medical School, UNITED STATES

## Abstract

The clinical relevance of cohesin in DNA repair, tumorigenesis, and severe birth defects continues to fuel efforts in understanding cohesin structure, regulation, and enzymology. Early models depicting huge cohesin rings that entrap two DNA segments within a single lumen are fading into obscurity based on contradictory findings, but elucidating cohesin structure amid a myriad of functions remains challenging. Due in large part to integrated uses of a wide range of methodologies, recent advances are beginning to cast light into the depths that previously cloaked cohesin structure. Additional efforts similarly provide new insights into cohesin enzymology: specifically, the discoveries of ATP-dependent transitions that promote cohesin binding and release from DNA. In combination, these efforts posit a new model that cohesin exists primarily as a relatively flattened structure that entraps only a single DNA molecule and that subsequent ATP hydrolysis, acetylation, and oligomeric assembly tether together individual DNA segments.

## Introduction

**Fig 1:**
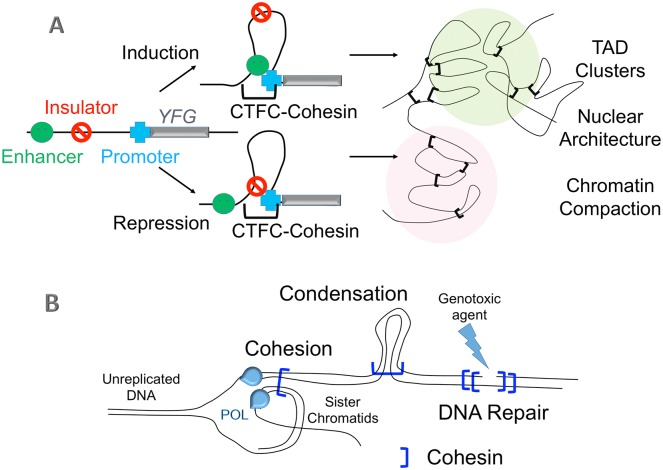
Cohesin functions. (A) DNA segment interactions stabilized by CTCF (transcriptional repressor) and cohesins define looped domains that aggregate into clusters of similar transcription outputs (active or silenced), termed topologically associated domains (TADs). TAD aggregation of both *cis* (with a single chromosome) and *trans* (involving two or more chromosomes) domains is critical for proper development and normal cell proliferation [1–3]. (B) DNA segment interactions stabilized by cohesins (independent of CTCF) during S phase are critical for meiotic and mitotic sister chromatid tethering, chromosome condensation, and DNA repair [4–6].

While simple in concept, the binding together of two or more DNA segments is critical to ensure human health. For instance, DNA interactions either at the base of a looped DNA molecule or between nonidentical chromosomes stabilize regulatory element (enhancers, promoters, insulators) registrations that deploy developmental transcription programs. Stabilized loops also compact and compartmentalize chromatin (Fig 1). DNA interactions between sister chromatids both identify chromatids as sisters to ensure high fidelity chromosome segregation and provide access to template DNA required for error-free repair of double-strand breaks (Fig 1).

Cohesins are protein complexes that contain Smc1, Smc3, Mcd1/Scc1/RAD21, SA1,2/Scc3, Pds5, and Sororin (in metazoan cells) and are critical for each of these DNA associations. Thus, cohesin pathway mutations that deregulate transcription programs produce severe and multispectrum birth defect maladies such as Roberts syndrome (RBS), Cornelia de Lange syndrome (CdLS), and Warsaw breakage syndrome (WBS) (Fig 1) [7–10]. Transcriptional deregulation is also likely to underlie the tight correlation between cohesin mutation and numerous forms of cancer that include aggressive melanoma, leukemia, and breast, astrocytic, and colorectal cancers [11–13]. Alternatively, cohesin mutations that abrogate tethering together of sister chromatids results in genotoxic sensitivity, aneuploidy (a hallmark of cancer cells), and apoptotic cell death (which may exacerbate birth defects through proliferative stem cell loss) [7,14–16]. Elucidating the structure through which cohesins bind together DNA segments thus remains of immense interest to both clinical and basic science researchers.

## Three Truths of Cohesin Structure

Early studies provided key insights into cohesin subunit orientations and binding interfaces, efforts augmented by recent crystallization studies [17–20]. Despite the crucial and multifaceted role for cohesins in human health, however, a thorough mechanistic view of cohesin's role in DNA tethering remains unclear. Interpreting results from even the most heroic of efforts must be tempered by three realities, or truths, that involve limited assemblies, distinct cohesin populations, and a bias toward predetermination.

### Truth #1: Limited Assemblies

**Fig 2:**
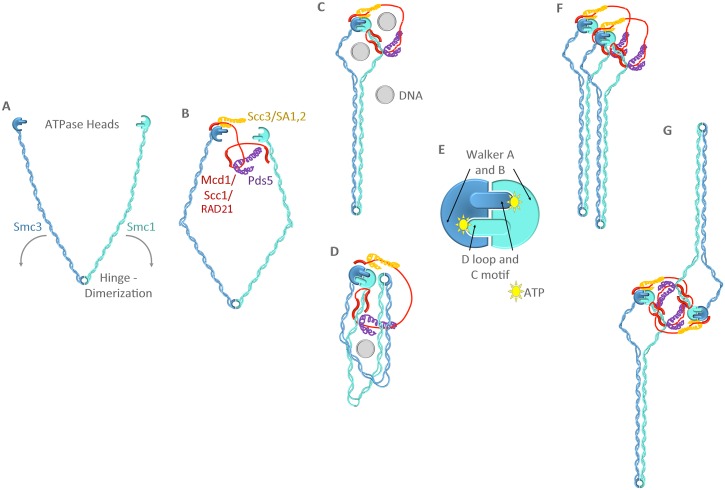
Stages of cohesin assembly. (A) Elongated coiled coil Smc1 and Smc3 proteins dimerize via hinge associations. (B) Smc1,3 become loosely tethered by Mcd1/Scc1/RAD21, which in turn recruits Pds5 and Scc3/SA1,2 (3 globular head structure). (C) and (D) Smc1,3 heads become tightly apposed and coiled coil domains zipper to form predominantly rodlike structures. Smc1,3 coiled coil domains can fold over into a C-clamp conformation to promote head–hinge association. Potential DNA entrapment sites are shown, but subunit dissociations (hinge–hinge, Smc1,3 ATPase heads, or Smc3-Mcd1/Scc1/Rad21) that allow entrapment, or whether there is a physiological role for C-clamp cohesins, remain hotly debated. (E) ATPase domains are composite structures that contain Walker A and B motifs from one Smc and D loop and C motif from the other Smc. (F) and (G) Hypothetical oligomerization models include intercohesin coiled coil or head–head binding, through which DNA segments (not shown) become tethered together. See text for references and further details.

The reality is that the methodologies through which cohesins are either assembled or enriched profoundly impact the apparent resulting cohesin structure (Fig 2). For instance, in vitro assembly of recombinant human Smc1,3 appears as an elongated “pair of cherries” in which the ATPase heads, depending on the hinge angle, are separated by the nearly 50 nm length of each Smc coiled coil domain [21]. In the presence of recombinant Mcd1/Scc1 and Scc3/SA1, Smc1,3 head domains become more proximally situated—but remain 25 nm apart [21]. Analyses of both human and *Xenopus* cohesins assembled in vivo but then extracted and enriched yield images of separate ATPase heads (similar to Smc1,3 heterodimers) or proximally associated heads but ones comprising two or three distinct globular domains [22]. In contrast, cohesins in living cells (analyzed by fluorescence resonance energy transfer [FRET] in the absence of extraction and enrichment) document exquisitely close Smc1,3 heads—3 nm apart. This distance is likely defined by the two ATP molecules tightly sandwiched between SMC1,3 heads [23,24]. The cautious interpretation is that images obtained of in vivo assemblies after extraction (including homogenization, high salt extraction, sonication, gel filtration, sedimentation, and/or immunochromatagraphy) likely capture stages of complex disassembly and/or disruption that mirror the de novo partial assemblies obtained using recombinant components.

### Truth #2: Distinct Cohesin Populations

**Fig 3:**
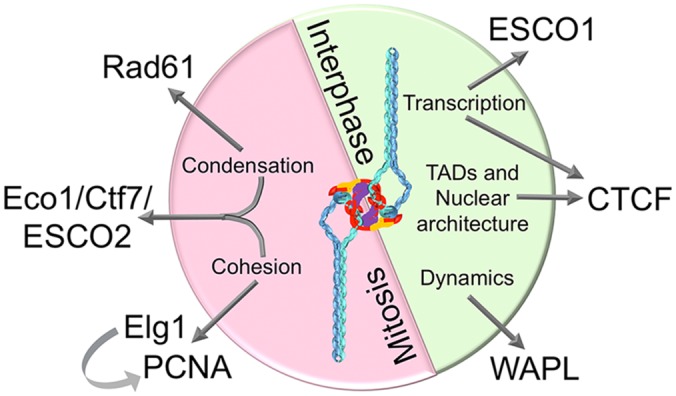
Multifaceted roles of cohesin. Cohesin functions are separable through cell cycle (red: mitotic; green: interphase) and genetic manipulations. For instance, mutation of *RAD61* suppresses only condensation defects, while mutation of *ELG1* (mimicking overexpression of PCNA) suppresses only cohesion defects otherwise present in mitotic cells deficient in cohesin activation. Note that human paralogs ESCO1,2 (yeast Eco1/Ctf7) and Rad61/WAPL orthologs exhibit predominantly separate functions. See text for references.

Should diversity of function (cohesion, condensation, DNA repair, transcription, etc.) lend a cautionary tale to a “one size fits all” notion of cohesin’s structural endpoint? Importantly, cohesin functions are demonstrably separable through specific regulatory factors that include ESCO1,2, ELG1, PCNA, Rad61/WAPL, CTCF, and Scc3/SA1,2 (Fig 3) [25–33]. If these regulatory factors produce cohesin subsets of dedicated and nonoverlapping function (including distinct binding partners and cell cycle specificities), then these cohesin “changlings” may exhibit unique endpoint conformations. Elucidating cohesin’s endpoint structure is further complicated by a diminishing fraction of functioning cohesins. For instance, over 50% of cohesins are soluble and thus unlikely to reflect a sister chromatid tethering state. Of the fraction of cohesins deposited during S phase, Smc3 is acetylated by Eco1/Ctf7 in a reaction limited to a postreplication fork context in order to participate in cohesion [34,35]. Experimental reduction of cohesin levels to less than 15% of wild-type results in retention of sister chromatid tethering [36], raising the possibility that only a small subset of chromatin-bound cohesins functionally participate in cohesion. It follows that the popular view of cohesin’s endpoint structure, one predicated on images obtained in an environment of “changling” populations and an overabundance of intermediates, warrants a measure of skepticism.

### Truth #3: Single Ring Predetermination

Cohesins are portrayed in the most current and prestigious publications as huge monomeric rings with near-perfect round lumens that entrap within two DNA segments. In contrast, however, are numerous findings that cohesins are relatively flattened structures that entrap only a single DNA segment, such that DNA tetherings rely on cohesin oligomerization (discussed in detail below). Is citing findings contrary to the notion of a huge ring that entraps two DNA segments an exercise in “cherry-picking”? The reality is that imaging studies of Smc complexes largely exclude analyses of higher-order complexes through sedimentation, filtration, and chromatography protocols that enrich for monomers [21,22,37]. Even so, both cohesin rods and oligomers are readily apparent in micrographs obtained using electron microscopy (EM) [21,37]. At issue then are the imposed criteria that exclude rods and oligomers and instead limit analyses solely to monomeric "complexes with clearly recognizable coiled coils" [21]—criteria that can provide no other result than open rings! Future efforts ultimately will establish whether it is the inclusion or exclusion of data that colors current views of cohesin structure, but enhanced resolve at the editorial level to include reviewers of disparate viewpoints would provide much needed balance. Below, I discuss new results that cohesins are relatively flattened structures before turning to evidence of cohesin oligomers.

## Cohesins: Rod or Ring?

How do we move toward a fully assembled (and functioning) endpoint of cohesin assembly (Fig 2)? A combination of EM, FRET, and bis-maleimidoethane (BMOE) cross-linking studies of evolutionarily conserved prokaryotic Smc-ScpAB complexes (*Bs*SMC and *Pf*Smc) document that Smc coiled coil domains “zipper” in an intermolecular fashion to form rods. EM imaging of SMC-related MukB (*Ec*MukBEF) similarly yields images of closely apposed (zippered rods) and separated (V or open) coiled coil conformations [37,38]. Cohesins from yeast predominantly yield EM images of coiled coil domains zippered up over a significant portion of the SMC complex to yield Y-type structures [39]. Are rods and Ys artifacts of preparation, or instead are flexible Vs and rings indicative of cohesin disassembly and/or disruption? Methodologies that complement EM-based techniques provide new and overwhelming evidence that the cohesin ring model—in which coiled coil arms are separated to form a huge 40 nm lumen—requires extensive revision. First, N-Hydroxysuccinimide ester linkages induced in closely apposed lysines in vivo reveal that a significant portion of human Smc1,3 coiled coil domains are very closely apposed (lysine cross-link distances below 6 nm!) [21]. Second, cohesin diffuses along DNA curtains (linear DNA tethered at both ends) past a 10 nm barrier but is blocked by a 20 nm barrier (DNA-tethered quantum dot). Further defining the cohesin lumen is that the 13 nm DNA translocase FtsK pushes cohesin along DNA instead of passing through the cohesin lumen [40]. Third, transmission electron microscopy (TEM) produces images of minichromosome sisters that appear anchored together by an extended solid rod roughly the length of flattened cohesin complexes that are largely devoid of a central lumen [41]. Fourth, small-angle X-ray scattering analyses of Smc1,3 proteins (hinge-truncated) reveal that coiled coils emerge from the ATPase heads in a parallel fashion [38]. These rodlike structures are similar to that of condensin (chromatin compacting complex that contains Smc2,4) and also prokaryotic Smc complexes. Indeed, almost universal evidence from EM, FRET, chemical cross-linking, and atomic force microscopy (AFM) documents rodlike condensin complexes [22,37,38,42,43]. A notable exception comes from liquid AFM performed on in vivo–assembled but extracted and purified yeast condensins [44]. In that study, condensins were predominantly folded over to promote hinge–head associations, but rings and lassos (dissociated heads in which only one appeared bound to the hinge) were also observed. Whether such intermediate structures are predicated on the dynamics of partially disrupted and flexible complexes (having survived extractions and enrichments) or represent functional cycles (despite conformation changes that occur in the absence of DNA and ATP) remains unknown [44]. Regardless, the similar degree of conservation among coiled coil domains within Smc1,3 cohesin, and Smc2,4 condensin family members is consistent with the notion that mutation is limited or constrained to preserve intermolecular binding along the entire coiled coil domain [45,46]. In summary, these findings herald a new view of cohesin as a relatively flattened rodlike monomer in which Smc1,3 coiled coils zipper to produce a lumen severely limited in size (<13 nm). Intermolecular coiled coil associations, however, may be conformationally plastic (beyond V and ring precursor assemblies) based on findings that sealing together Smc1,3 hinges in either yeast or human precludes DNA entrapment [47,48].

## Cohesins: Evidence for Oligomers

A second step in moving toward a functional cohesin endpoint structure is determining the mechanism by which cohesins tether together two or more DNA segments. Is there an oligomeric state of cohesin assembly? EM analyses describe oligomers of MukB and BsSMC as rosettes of ordered SMC assemblies [37], although oligomers are unfortunately largely excluded from EM analyses [21,37,39]. Additional lines of evidence document not only intracohesin zippering (Smc1-Smc3 coiled coils described above) but also intercohesin oligomerization (zippering between neighboring Smc1,3 complexes). For instance, TEM images of tethered sister minichromosomes include rods that are wider than a single flattened cohesin complex, suggesting that cohesins oligomerize side by side [41]. Higher-order coiled coil assemblies are strongly supported by numerous AFM studies in which both cohesins and condensins clearly fold back on themselves (Fig 2), likely stabilized through tetrameric intermolecular coiled coil associations to produce complexes of roughly 25 nm in length with heads and hinges closely apposed [43,44,49]. Biochemical and cytological analyses document both Pds5 and SA1/Scc3 binding proximal to both ATPase head and hinge domains, consistent with cohesin folding and intermolecular coiled coil associations [21,23]. Crystal structures further reveal that N-terminal Mcd1 helices associate directly with the head-proximal coiled coil domain of Smc3 [18], extending the role of SMC coiled coil interactions to include other helices and coiled domains.

Beyond intermolecular coiled coil zippering, other findings support cohesin oligomerization. Biochemical studies that documented cohesin subunit interactions concomitantly provided early evidence that Mcd1/Scc1 may indeed cross-link separate Smc1,3 complexes: HA-tagged Smc3 coimmunoprecipitates HIS-tagged Smc3 in an Mcd1/Scc1-dependent process [39]. Independent studies further support oligomerization through Mcd1/Scc1 associations: FLAG-tagged Mcd1/Scc1 coimmunoprecipitates both endogenous and MYC-tagged Mcd1/Scc1 [50]. Another driver of oligomeric assembly may derive from SMC head domain dimerizations. For instance, Smc1 ATPase head domains homodimerize, an oligomerization mechanism supported by crystallization studies that human condensin Smc2 head domains also homodimerize [51,52]. Additional oligomerization strategies include bracelets and duplexes (Smc1 ATPase head or hinge from one cohesin binds Smc3 ATPase head or hinge from a neighboring cohesin, respectively) and ring concatenations (handcuffs) [53]. The numerous oligomerization mechanisms described above—intercohesin coiled coil zippering, Mcd1 bridging of juxtaposed cohesins, homotypic ATPase head domain associations, bracelets, duplexes, and concatenations—are not mutually exclusive, such that combinations may help impose cell cycle and function-specific conformations (Fig 2).

The cohesin oligomerization model makes two crucial predications that distinguish it from the simplistic single ring entrapment model. The first of these is that oligomerization allows for interallelic complementation, such that coexpression of two mutant alleles that individually fail to support viability might now provide for cell growth. In fact, the combination of *mcd1-Q266* and *mcd1-1* alleles fully rescues the condensation, cohesion, and cell viability defects exhibited by cells under conditions in which either single mutation alone is both lethal and elicits dramatic cohesion and condensation defects. Moreover, coexpression of *smc3-42* and *smc3-K113R* mutations similarly support robust cell viability under conditions in which either single allele is lethal [54]. Importantly, expression of either mcd1-Q266 or smc3-K113R subunit restores chromatin binding of the cognate mcd1-1 and smc3-42 subunit, in further support of the model that these interallelic complementations are predicated on intimately juxtaposed cohesin complexes [54]. The second prediction of the oligomeric tethering model is that cohesion inactivation (leading to separation of previously tethered sister chromatids) can be distinct from cohesin release from DNA. In contrast, the single ring around two sister chromatids model requires that separation of previously tethered sisters must occur through cohesin release from DNA. When this prediction was tested directly, mitotic inactivation of cohesion (using a temperature-sensitive Pds5 allele) resulted in sister separation despite nearly full retention of chromatin-bound cohesin [33,55]. Identical results were obtained during characterization of *mcd1-Q266Q* mutant cells [56]. Importantly, chromatin-retained cohesins in *pds5*-inactivated cells retain their acetylation state (a modification that occurs only during S phase), negating arguments that the detected cohesins involved newly loaded (i.e., during mitosis) complexes [33]. This reemerging model of cohesion establishment—that cohesins stably deposited onto each sister chromatid are subsequently converted to a tethering-competent, higher-order structure [57]—suggests that sister chromatid tethering requires multiple transition states that are likely regulated by both cohesin ATP hydrolysis and Eco1/Ctf7-dependent acetylation.

## Cohesin Enzymology: Is It Only about Gates?

Cohesins are actually quite dynamic—an attribute required for distinct functions throughout the cell cycle (Fig 3). In metazoan cells, cohesin deposition and release cycles start late in mitosis of the previous cell cycle and continue through G1 to provide for adaptive regulation of transcriptional programs and nuclear architecture. Similar to just about every other chromatin-associated protein complex, these cohesins are stripped from chromatin during passage of the DNA replication fork [58,59]—a process that allows for genome-wide resetting of transcriptional outputs. In turn, deposition during S phase ensures cohesin decoration onto each sister chromatid, with subsequent Eco1/Ctf7-dependent acetylation of Smc3 engendering both Sororin recruitment (metazoan cells) and sister chromatid tethering [60]. Deposition extends into mitosis (although these cohesins normally do not participate in cohesion)—after which chromatid-bound cohesins are inactivated by proteolysis—defining anaphase onset and resulting in chromosome segregation. Intriguingly, a large fraction of chromatid-bound cohesins are removed during prophase in a proteolytic-independent process that requires SA1,2/Scc3 phosphorylation [35,61]. Identifying the “gate” or subunit pair that regulates cohesin dynamics, however, is obfuscated by conflicting evidence and potentially distinct entry and exit reactions [47,62,63], reminding us that we remain in the early stages of cohesin research. Regardless, elucidating the enzymology of cohesins in different parts of the cell cycle is vital to resolve transition states through which both adaptable and stable cohesin populations are simultaneously achieved. Cohesin enzymology is complex, however, because each of the two ATPase domains are composite structures (Fig 2). For instance, the Smc3 ATPase requires Walker A and B motifs from Smc3 as well as the D-loop and Signature or C-motif from Smc1 [64,65]. Thus, mutations in Smc1 can abrogate Smc3 ATP hydrolysis and vice versa. Moreover, Smc1,3 are asymmetrically positioned within the cohesin complex, and individual ATP hydrolysis cycles appear similarly asymmetric in terms of cohesin function [18,21,62,63,66]. Thus, how ATP binding and hydrolysis and acetylation impact deposition and stability remain exciting frontiers in cohesin research.

### A New Role for ATP Hydrolysis

Scc2/Mis4/NIPBL and Scc4/Ssl3/MAU-2 (herein Scc2,4) are required for robust deposition of cohesin onto DNA and stimulates Smc1,3 ATP hydrolysis. In turn, Smc1,3 head domains that can both bind and hydrolyze ATP are required for stable cohesin deposition onto DNA [67–71]. In the limited context of a single ring entrapment model, these early reports suggested that Scc2,4 promotes ATP hydrolysis (and ADP dissociation) to open the cohesin ring such that subsequent ATP binding closes the ring to stably entrap within both sister chromatids. As previously pointed out, however [65], this model requires cessation of ATP hydrolysis to maintain cohesion through mitosis.

Recent findings document a more complex series of transition states. For instance, chromatin-associated cohesins exhibit at least two different residency states—one dynamic (sensitive to elevated salt levels) and a second that is quite stable (insensitive to elevated salt levels). To participate in sister chromatid tethering, however, chromatin-bound cohesins must be acetylated by Eco1/Ctf7 [56,72–81]. If sister chromatid tethering is predicated on cohesin oligomerization, might ATP hydrolysis both persist after stable cohesin deposition and facilitate Eco1/Ctf7 cohesion establishment? In directly testing the first of these predictions, the Koshland lab discovered that robust ATP hydrolysis indeed persists in stably chromatin-bound cohesins [65]. The second prediction that Smc1,3 ATP hydrolysis may promote tethering in conjunction with Eco1/Ctf7-dependent acetylation of Smc3 was borne out by identification of *SMC1* mutations (such as *smc1-D1164E*) that bypass Eco1/Ctf7 function. Intriguingly, smc1-D1164E supports cohesin deposition onto DNA—but abrogates Smc3 ATP hydrolysis [63,65]. How might this modified subunit promote cohesion in the absence of Eco1/Ctf7? Possibilities include that this *smc1* allele mimics Smc3 acetylation and/or locks Smc3 in an intermediate ATP hydrolysis (ADP+Pi) state that promotes cohesion. The notion, however, that Smc3 ATP hydrolysis is blocked by Eco1/Ctf7 acetylation contrasts earlier findings [79] and is further challenged by the fact that *smc1-D1164E eco1/ctf7* double-mutant strains still exhibit significant cohesion defects, albeit well below the level of *eco1/ctf7* single-mutant cohesion defects [63,65]. The degree to which SMC ATP hydrolysis ceases after establishment thus requires further testing.

The identification of smc1-D1164E may provide insights regarding the putative mechanism of cohesin oligomerization. Given that *smc1-D1164E* at least partially rescues the cohesion defect otherwise present in *eco1/ctf7* mutant cells, smc1-D1164E may support not only cohesin binding to DNA but also intermolecular associations required for oligomerization. The determining role for Smc3 acetylation or ATP hydrolysis in sister chromatid tethering through oligomerization thus remains an important question. Intriguingly, *smc1A-L1128V* (analogous to yeast smc1-L1129V that bypasses Eco1/Ctf7 function despite loss of ATPase activity) expression in human cells produces unresolved sister chromatids [63]—a phenotype typically produced by defects in the prophase-specific removal of cohesins from chromosome arms. Further analyses of ATPase mutations that appear capable of separating cohesin deposition from its nonproteolytic removal may thus prove quite valuable. Moving forward, in vitro systems that more faithfully mimic in vivo deposition (cohesin deposition that is stimulated by Scc2,4, ATP, and DNA, is salt-resistant, and is targeted to previously identified cohesin-associated DNA loci) and similar physiologically relevant assays, coupled with the incredible AFM DREEM imaging system (in which topological contributions by DNA and protein are resolvable), should provide exquisite specificity for future analyses of both cohesin ATP cycles and cohesin structure [65,79–82].

## Conclusion

In the 1997 movie *Men in Black*, Agent Kay (played by Tommy Lee Jones) consoles a recruit shaken by the revelation of aliens on earth. “Fifteen hundred years ago, everybody knew the earth was the center of the universe. Five hundred years ago, everybody knew the earth was flat and fifteen minutes ago, you knew that people were alone on this planet. Imagine what you'll know … tomorrow.” By analogy: fourteen years ago, everybody knew that the DNA replication fork passed through single huge cohesin rings previously loaded during G1 [39]. Ten years ago, everybody knew Smc1,3 ATP hydrolysis drove hinge dissociations that allowed for the capture of sister chromatids within a single lumen [47], and last year, everybody knew that cohesins captured DNA through Smc3 head-Mcd1 release and reclosure [62]. Given that flattened cohesins individually decorate sister chromatids during S phase and that tethering likely requires ATP and/or acetylation-dependent oligomerization, imagine what we will learn … tomorrow.

## References

[1] Merkenschlager M, Nora EP. CTCF and Cohesin in Genome Folding and Transcriptional Gene Regulation. Annu Rev Genomics Hum Genet. 2016;17: 8.1–8.27. 10.1146/annurev-genom-083115-022339 27089971

[2] Ghirlando R, Felsenfeld G. CTCF: making the right connections. Genes Dev. 2016;30: 881–891. 10.1101/gad.277863.116 27083996 PMC4840295

[3] Rudan M Vietri, Hadjur S. Genetic Tailors: CTCF and Cohesin Shape the Genome During Evolution. Trends Genet. 2015;31: 651–660. 10.1016/j.tig.2015.09.004 26439501

[4] Skibbens RV, Colquhoun JM, Green MJ, Molnar CA, Sin DN, Sullivan BJ, Tanzosh EE. Cohesinopathies of a feather flock together. PLoS Genet 2013;9: e1004036 10.1371/journal.pgen.1004036 24367282 PMC3868590

[5] Rankin S. Complex elaboration: making sense of meiotic cohesin dynamics. FEBS J. 2015;282: 2426–2443. 10.1111/febs.13301 25895170 PMC4490075

[6] Hirano T. Chromosome Dynamics during Mitosis. Cold Spring Harb Perspect Biol. 2015;7: a015792 10.1101/cshperspect.a015792 25722466 PMC4448609

[7] Cucco F, Musio A. Genome stability: What we have learned from cohesinopathies. Am J Med Genet C Semin Med Genet. 2016;172: 171–178. 10.1002/ajmg.c.31492 27091086

[8] Dorsett D, Merkenschlager M. Cohesin at active genes: a unifying theme for cohesin and gene expression from model organisms to humans. Curr Opin Cell Biol. 2013;25: 327–333. 10.1016/j.ceb.2013.02.003 23465542 PMC3691354

[9] Banerji R, Eble DM, Iovine MK, Skibbens RV. Esco2 regulates cx43 expression during skeletal regeneration in the zebrafish fin. Dev Dyn. 2016;245: 7–21. 10.1002/dvdy.24354 26434741

[10] Liu J, Krantz ID. Cornelia de Lange syndrome, cohesin, and beyond. Clin Genet. 2009;76: 303–314. 10.1111/j.1399-0004.2009.01271.x 19793304 PMC2853897

[11] Mannini L, Musio A. (2011) The dark side of cohesin: the carcinogenic point of view. Mutat Res 728: 81–87. 22106471 10.1016/j.mrrev.2011.07.004

[12] Rhodes JM, McEwan M, Horsfield JA. Gene regulation by cohesin in cancer: is the ring an unexpected party to proliferation? Mol Cancer Res 2011;9: 1587–1607. 10.1158/1541-7786.MCR-11-0382 21940756

[13] Williams MS, Somervaille TC. Leukemogenic Activity of Cohesin Rings True. Cell Stem Cell 2015;17: 642–644. 10.1016/j.stem.2015.11.008 26637939

[14] Panigrahi AK, Pati D. Road to the crossroads of life and death: linking sister chromatid cohesion and separation to aneuploidy, apoptosis and cancer. Crit Rev Oncol Hematol. 2009;72: 181–193. 10.1016/j.critrevonc.2008.12.002 19162508 PMC2783576

[15] Horsfield JA, Print CG, Mönnich M. Diverse developmental disorders from the one ring: distinct molecular pathways underlie the cohesinopathies. Front Genet. 2012;3: 171 10.3389/fgene.2012.00171 22988450 PMC3439829

[16] Rudra S, Skibbens RV. Cohesin codes - interpreting chromatin architecture and the many facets of cohesin function. J Cell Sci 2013;126: 31–41. 10.1242/jcs.116566 23516328 PMC3603509

[17] Roig MB, Löwe J, Chan KL, Beckouët F, Metson J, Nasmyth K. Structure and function of cohesin's Scc3/SA regulatory subunit. FEBS Lett. 2014;588: 3692–3702. 10.1016/j.febslet.2014.08.015 25171859 PMC4175184

[18] Gligoris TG, Scheinost JC, Bürmann F, Petela N, Chan KL, Uluocak P, et al Closing the cohesin ring: structure and function of its Smc3-kleisin interface. Science 2014;346: 963–967. 10.1126/science.1256917 25414305 PMC4300515

[19] Lee BG, Roig MB, Jansma M, Petela N, Metson J, Nasmyth K, Löwe J. Crystal Structure of the Cohesin Gatekeeper Pds5 and in Complex with Kleisin Scc1. Cell Rep. 2016;14: 2108–2115. 10.1016/j.celrep.2016.02.020 26923598 PMC4793087

[20] Muir KW, Kschonsak M, Li Y, Metz J, Haering CH, Panne D. Structure of the Pds5-Scc1 Complex and Implications for Cohesin Function. Cell Rep. 2016;14: 2116–2126. 10.1016/j.celrep.2016.01.078 26923589

[21] Huis in 't Veld PJ, Herzog F, Ladurner R, Davidson IF, Piric S, Kreidl E, et al Characterization of a DNA exit gate in the human cohesin ring. Science. 2014;346: 968–972. 10.1126/science.1256904 25414306

[22] Anderson DE, Losada A, Erickson HP, Hirano T. Condensin and cohesin display different arm conformations with characteristic hinge angles. J Cell Biol. 2002;156: 419–424. 10.1083/jcb.200111002 11815634 PMC2173330

[23] Mc Intyre J, Muller EG, Weitzer S, Snydsman BE, Davis TN, Uhlmann F. In vivo analysis of cohesin architecture using FRET in the budding yeast Saccharomyces cerevisiae. EMBO J. 2007;26: 3783–3793. 10.1038/sj.emboj.7601793 17660750 PMC1952217

[24] Lammens A, Schele A, Hopfner KP. Structural biochemistry of ATP-driven dimerization and DNA-stimulated activation of SMC ATPases. Curr Biol. 2004;14: 1778–1782. 15458651 10.1016/j.cub.2004.09.044

[25] Skibbens RV, Corson LB, Koshland D, Hieter P. Ctf7p is essential for sister chromatid cohesion and links mitotic structure to the DNA replication machinery. Genes Dev 1999;13: 307–319. 9990855 10.1101/gad.13.3.307 PMC316428

[26] Maradeo ME, Skibbens RV. The Elg1-RFC clamp-loading complex performs a role in sister chromatid cohesion. PLoS ONE 2009:4: e4707 10.1371/journal.pone.0004707 19262753 PMC2650802

[27] Parnas O, Zipin-Roitman A, Mazor Y, Liefshitz B, Ben-Aroya S, Kupiec M. The ELG1 clamp loader plays a role in sister chromatid cohesion. PLoS ONE 2009;4: e5497 10.1371/journal.pone.0005497 19430531 PMC2676507

[28] Guacci V., and Koshland D. Cohesin-independent segregation of sister chromatids in budding yeast. Mol Biol Cell 2012;23: 729–739. 10.1091/mbc.E11-08-0696 22190734 PMC3279399

[29] Orgil O, Matityahu A, Eng T, Guacci V, Koshland D, Onn I. A conserved domain in the scc3 subunit of cohesin mediates the interaction with both mcd1 and the cohesin loader complex. PLoS Genet. 2015;11: e1005036 10.1371/journal.pgen.1005036 25748820 PMC4352044

[30] Rahman S, Jones MJ, Jallepalli PV. Cohesin recruits the Esco1 acetyltransferase genome wide to repress transcription and promote cohesion in somatic cells. Proc Natl Acad Sci U S A. 2015;112: 11270–11275. 10.1073/pnas.1505323112 26305936 PMC4568707

[31] Rowland BD, Roig MB, Nishino T, Kurze A, Uluocak P, Mishra A, et al Building sister chromatid cohesion: smc3 acetylation counteracts an antiestablishment activity. Mol Cell 2009;22: 763–774. 10.1016/j.molcel.2009.02.028 19328069

[32] Sutani T, Kawaguchi T, Kanno R, Itoh T, Shirahige K. Budding yeast Wpl1(Rad61)-Pds5 complex counteracts sister chromatid cohesion-establishing reaction. Curr Biol 2009;19: 492–497. 10.1016/j.cub.2009.01.062 19268589

[33] Tong K, Skibbens RV. Pds5 regulators segregate cohesion and condensation pathways in Saccharomyces cerevisiae. Proc Natl Acad Sci U S A 2015;112: 7021–7026. 10.1073/pnas.1501369112 25986377 PMC4460518

[34] Skibbens RV, Maradeo M, Eastman L. Fork it over: the cohesion establishment factor Ctf7p and DNA replication. J Cell Sci. 2007;120: 2471–2477. 17646671 10.1242/jcs.011999

[35] Onn I, Heidinger-Pauli JM, Guacci V, Unal E, Koshland DE. Sister chromatid cohesion: a simple concept with a complex reality. Annu Rev Cell Dev Biol. 2008;24: 105–129. 10.1146/annurev.cellbio.24.110707.175350 18616427

[36] Heidinger-Pauli JM, Mert O, Davenport C, Guacci V, Koshland D. Systematic reduction of cohesin differentially affects chromosome segregation, condensation, and DNA repair. Curr Biol. 2010;20: 957–963. 10.1016/j.cub.2010.04.018 20451387 PMC2892909

[37] Melby TE, Ciampaglio CN, Briscoe G, Erickson HP. The symmetrical structure of structural maintenance of chromosomes (SMC) and MukB proteins: long, antiparallel coiled coils, folded at a flexible hinge. J Cell Biol. 1998;142: 1595–1604. 9744887 10.1083/jcb.142.6.1595 PMC2141774

[38] Soh YM, Bürmann F, Shin HC, Oda T, Jin KS, Toseland CP, et al Molecular basis for SMC rod formation and its dissolution upon DNA binding. Mol Cell. 2015;57: 290–303. 10.1016/j.molcel.2014.11.023 25557547 PMC4306524

[39] Haering CH, Löwe J, Hochwagen A, Nasmyth K. Molecular architecture of SMC proteins and the yeast cohesin complex. Mol Cell. 2002;9: 773–788. 11983169 10.1016/s1097-2765(02)00515-4

[40] Stigler J, Çamdere GÖ, Koshland DE, Greene EC. Single-Molecule Imaging Reveals a Collapsed Conformational State for DNA-Bound Cohesin. Cell Rep. 2016;15: 988–998. 10.1016/j.celrep.2016.04.003 27117417 PMC4856582

[41] Surcel A, Koshland D, Ma H, Simpson RT. Cohesin interaction with centromeric minichromosomes shows a multi-complex rod-shaped structure. PLoS ONE. 2008;3: e2453 10.1371/journal.pone.0002453 18545699 PMC2408725

[42] Barysz H, Kim JH, Chen ZA, Hudson DF, Rappsilber J, Gerloff DL, Earnshaw WC. Three-dimensional topology of the SMC2/SMC4 subcomplex from chicken condensin I revealed by cross-linking and molecular modelling. Open Biol. 2015;5: 150005 10.1098/rsob.150005 25716199 PMC4345284

[43] Yoshimura SH, Hizume K, Murakami A, Sutani T, Takeyasu K, Yanagida M. Condensin architecture and interaction with DNA: regulatory non-SMC subunits bind to the head of SMC heterodimer. Curr Biol. 2002;12: 508–513. 11909539 10.1016/s0960-9822(02)00719-4

[44] Eeftens JM, Katan AJ, Kschonsak M, Hassler M, de Wilde L, Dief EM, et al Condensin Smc2-Smc4 Dimers Are Flexible and Dynamic. Cell Rep. 2016;14: 1813–1818. 10.1016/j.celrep.2016.01.063 26904946 PMC4785793

[45] White GE, Erickson HP. Sequence divergence of coiled coils--structural rods, myosin filament packing, and the extraordinary conservation of cohesins. J Struct Biol. 2006;154: 111–121. 10.1016/j.jsb.2006.01.001 16495084

[46] White GE, Erickson HP. The coiled coils of cohesin are conserved in animals, but not in yeast. PLoS ONE. 2009;4: e4674 10.1371/journal.pone.0004674 19262687 PMC2650401

[47] Gruber S, Arumugam P, Katou Y, Kuglitsch D, Helmhart W, Shirahige K, Nasmyth K. Evidence that loading of cohesin onto chromosomes involves opening of its SMC hinge. Cell. 2006;127: 523–537. 10.1016/j.cell.2006.08.048 17081975

[48] Johannes Buheitel J, Stemmann O. Prophase pathway-dependent removal of cohesin from human chromosomes requires opening of the Smc3-Scc1 gate. EMBO J. 2013;32: 666–676. 10.1038/emboj.2013.7 23361318 PMC3590994

[49] Sakai A, Hizume K, Sutani T, Takeyasu K, Yanagida M. Condensin but not cohesin SMC heterodimer induces DNA reannealing through protein-protein assembly. EMBO J. 2003;22: 2764–2775. 10.1093/emboj/cdg247 12773391 PMC156744

[50] Zhang N, Kuznetsov SG, Sharan SK, Li K, Rao PH, Pati D. A handcuff model for the cohesin complex. J Cell Biol. 2008;183: 1019–1031. 10.1083/jcb.200801157 19075111 PMC2600748

[51] Haering CH, Schoffnegger D, Nishino T, Helmhart W, Nasmyth K, Löwe. Structure and stability of cohesin's Smc1-kleisin interaction. Mol Cell. 2004;15: 951–964. 10.1016/j.molcel.2004.08.030 15383284

[52] Uchiyama S, Kawahara K, Hosokawa Y, Fukakusa S, Oki H, Nakamura S, et al Structural Basis for Dimer Formation of Human Condensin Structural Maintenance of Chromosome Proteins and Its Implications for Single-stranded DNA Recognition. J Biol Chem. 2015;290: 29461–29477. 10.1074/jbc.M115.670794 26491021 PMC4705948

[53] Huang CE, Milutinovich M, Koshland D. Rings, bracelet or snaps: fashionable alternatives for Smc complexes. Philos Trans R Soc Lond B Biol Sci. 2005;360: 537–542. 10.1098/rstb.2004.1609 15897179 PMC1569475

[54] Eng T, Guacci V, Koshland D. Interallelic complementation provides functional evidence for cohesin-cohesin interactions on DNA. Mol Biol Cell. 2015;26: 4224–4235. 10.1091/mbc.E15-06-0331 26378250 PMC4642856

[55] Kulemzina I, Schumacher MR, Verma V, Reiter J, Metzler J, Failla AV, et al Cohesin rings devoid of Scc3 and Pds5 maintain their stable association with the DNA. PLoS Genet. 2012;8: e1002856 10.1371/journal.pgen.1002856 22912589 PMC3415457

[56] Eng T, Guacci V, Koshland D. ROCC, a conserved region in cohesin's Mcd1 subunit, is essential for the proper regulation of the maintenance of cohesion and establishment of condensation. Mol Biol Cell. 2014;25: 2351–2364. 10.1091/mbc.E14-04-0929 24966169 PMC4142609

[57] Skibbens RV. Holding your own: establishing sister chromatid cohesion. Genome Res. 2000;10: 1664–1671. 11076851 10.1101/gr.153600

[58] Rudra S, Skibbens RV. Chl1 DNA helicase regulates Scc2 deposition specifically during DNA-replication in Saccharomyces cerevisiae. PLoS ONE. 2013;8: e75435 10.1371/journal.pone.0075435 24086532 PMC3784445

[59] Brüning JG, Howard JL, McGlynn P. Accessory replicative helicases and the replication of protein-bound DNA. J Mol Biol. 2014;426: 3917–3928. 10.1016/j.jmb.2014.10.001 25308339

[60] Peters JM, Nishiyama T. Sister chromatid cohesion. Cold Spring Harb Perspect Biol. 2012;4: a011130 10.1101/cshperspect.a011130 23043155 PMC3536341

[61] Murayama Y, Uhlmann F. Chromosome segregation: how to open cohesin without cutting the ring? EMBO J. 2013;32: 614–616. 10.1038/emboj.2013.22 23395901 PMC3590991

[62] Murayama Y, Uhlmann F. DNA Entry into and Exit out of the Cohesin Ring by an Interlocking Gate Mechanism. Cell. 2015;163: 1628–1640. 10.1016/j.cell.2015.11.030 26687354 PMC4701713

[63] Elbatsh AM, Haarhuis JH, Petela N, Chapard C, Fish A, Celie PH, et al Cohesin Releases DNA through Asymmetric ATPase-Driven Ring Opening. Mol Cell. 2016;61: 575–588. 10.1016/j.molcel.2016.01.025 26895426 PMC4769319

[64] Hopfner KP, Tainer JA. Rad50/SMC proteins and ABC transporters: unifying concepts from high-resolution structures. Curr Opin Struct Biol. 2003;13: 249–255. 12727520 10.1016/s0959-440x(03)00037-x

[65] Çamdere G, Guacci V, Stricklin J, Koshland D. The ATPases of cohesin interface with regulators to modulate cohesin-mediated DNA tethering. Elife. 2015;4: e11315 10.7554/eLife.11315 26583750 PMC4709263

[66] Beckouët F, Srinivasan M, Roig MB, Chan KL, Scheinost JC, Batty P, Hu B, Petela N, Gligoris T, Smith AC, Strmecki L, Rowland BD, Nasmyth K. Releasing Activity Disengages Cohesin's Smc3/Scc1 Interface in a Process Blocked by Acetylation. Mol Cell. 2016;61(4): 563–574. 10.1016/j.molcel.2016.01.026 26895425 PMC4769318

[67] Ciosk R, Shirayama M, Shevchenko A, Tanaka T, Toth A, Shevchenko A, Nasmyth K. Cohesin's binding to chromosomes depends on a separate complex consisting of Scc2 and Scc4 proteins. Mol Cell. 2000;5: 243–254. 10882066 10.1016/s1097-2765(00)80420-7

[68] Arumugam P, Gruber S, Tanaka K, Haering CH, Mechtler K, Nasmyth K. ATP hydrolysis is required for cohesin's association with chromosomes. Curr Biol. 2003;13: 1941–1953. 14614819 10.1016/j.cub.2003.10.036

[69] Arumugam P, Nishino T, Haering CH, Gruber S, Nasmyth K. Cohesin's ATPase activity is stimulated by the C-terminal Winged-Helix domain of its kleisin subunit. Curr Biol. 2006;16: 1998–2008. 10.1016/j.cub.2006.09.002 17055978

[70] Gillespie PJ, Hirano T. Scc2 couples replication licensing to sister chromatid cohesion in Xenopus egg extracts. Curr Biol. 2004;14: 1598–1603. 10.1016/j.cub.2004.07.053 15341749

[71] Watrin E, Schleiffer A, Tanaka K, Eisenhaber F, Nasmyth K, Peters JM. Human Scc4 is required for cohesin binding to chromatin, sister-chromatid cohesion, and mitotic progression. Curr Biol. 2006;16: 863–874. 10.1016/j.cub.2006.03.049 16682347

[72] Tóth A, Ciosk R, Uhlmann F, Galova M, Schleiffer A, Nasmyth K. Yeast cohesin complex requires a conserved protein, Eco1p(Ctf7), to establish cohesion between sister chromatids during DNA replication. Genes Dev. 1999;13: 320–333. 9990856 10.1101/gad.13.3.320 PMC316435

[73] Skibbens RV, Corson LB, Koshland D, Hieter P. Ctf7p is essential for sister chromatid cohesion and links mitotic chromosome structure to the DNA replication machinery. Genes Dev. 1999;13: 307–319. 9990855 10.1101/gad.13.3.307 PMC316428

[74] Milutinovich M, Unal E, Ward C, Skibbens RV, Koshland D. A multi-step pathway for the establishment of sister chromatid cohesion. PLoS Genet. 2007;3: e12 10.1371/journal.pgen.0030012 17238288 PMC1779304

[75] Gause M, Misulovin Z, Bilyeu A, Dorsett D. Dosage-sensitive regulation of cohesin chromosome binding and dynamics by Nipped-B, Pds5, and Wapl. Mol Cell Biol. 2010;30: 4940–4951. 10.1128/MCB.00642-10 20696838 PMC2950535

[76] Gerlich D, Koch B, Dupeux F, Peters JM, Ellenberg J. Live-cell imaging reveals a stable cohesin-chromatin interaction after but not before DNA replication. Curr Biol. 2006;16: 1571–1578. 10.1016/j.cub.2006.06.068 16890534

[77] Hu B, Itoh T, Mishra A, Katoh Y, Chan KL, Upcher W, Godlee C, Roig MB, Shirahige K, Nasmyth K.ATP hydrolysis is required for relocating cohesin from sites occupied by its Scc2/4 loading complex. Curr Biol. 2011;21: 12–24. 10.1016/j.cub.2010.12.004 21185190 PMC4763544

[78] Guacci V, Stricklin J, Bloom MS, Guō X, Bhatter M, Koshland D. A novel mechanism for the establishment of sister chromatid cohesion by the ECO1 acetyltransferase. Mol Biol Cell. 2015;26: 117–133. 10.1091/mbc.E14-08-1268 25378582 PMC4279223

[79] Ladurner R, Bhaskara V, Huis in 't Veld PJ, Davidson IF, Kreidl E, Petzold G, Peters JM. Cohesin's ATPase activity couples cohesin loading onto DNA with Smc3 acetylation. Curr Biol. 2014;24: 2228–2237. 10.1016/j.cub.2014.08.011 25220052 PMC4188815

[80] Onn I, Koshland D. In vitro assembly of physiological cohesin/DNA complexes. Proc Natl Acad Sci U S A. 2011;108: 12198–12205. 10.1073/pnas.1107504108 21670264 PMC3145678

[81] Murayama Y, Uhlmann F. Biochemical reconstitution of topological DNA binding by the cohesin ring. Nature. 2014;505: 367–371. 10.1038/nature12867 24291789 PMC3907785

[82] Wu D, Kaur P, Li ZM, Bradford KC, Wang H, Erie DA. Visualizing the Path of DNA through Proteins Using DREEM Imaging. Mol Cell. 2016;61: 315–323. 10.1016/j.molcel.2015.12.012 26774284 PMC5242388

