# Correction: Of Rings and Rods: Regulating Cohesin Entrapment of DNA to Generate Intra- and Intermolecular Tethers

**DOI:** 10.1371/journal.pgen.1006478

**Published:** 2016-12-01

**Authors:** Robert V. Skibbens

The protein CTCF is incorrectly labelled in Fig 1A as CTFC. Please view the correct Fig 1 here, which is also modified to include in Fig 1A CTCF-Cohesins that tether together topologically associated domains between chromosomes.

**Fig 1:**
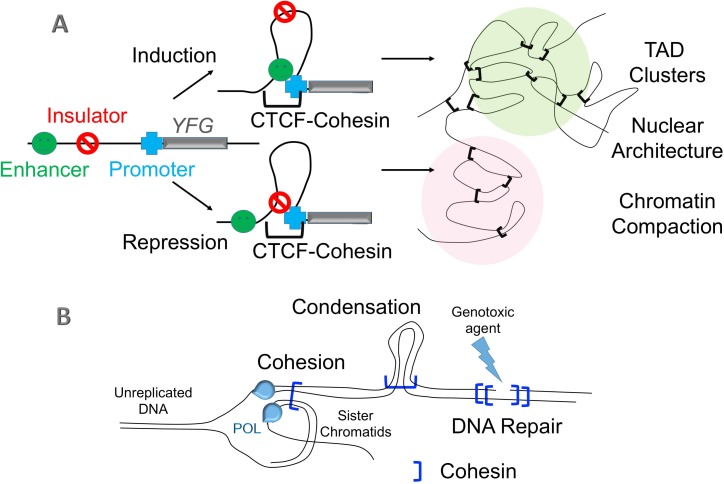
Cohesin functions. (A) DNA segment interactions stabilized by CTCF (transcriptional repressor) and cohesins define looped domains that aggregate into clusters of similar transcription outputs (active or silenced), termed topologically associated domains (TADs). TAD aggregation of both *cis* (with a single chromosome) and *trans* (involving two or more chromosomes) domains is critical for proper development and normal cell proliferation [1–3]. (B) DNA segment interactions stabilized by cohesins (independent of CTCF) during S phase are critical for meiotic and mitotic sister chromatid tethering, chromosome condensation, and DNA repair [4–6].
